# Serum TSH level stability after 5 years in euthyroid adults at low risk for thyroid dysfunction

**DOI:** 10.20945/2359-3997000000037

**Published:** 2018-05-07

**Authors:** Pedro Weslley Rosario, Maria Regina Calsolari

**Affiliations:** 1 Santa Casa de Misericórdia de Belo Horizonte Santa Casa de Belo Horizonte Programa de Pós-Graduação e Serviço de Endocrinologia Belo Horizonte MG Brasil Programa de Pós-Graduação e Serviço de Endocrinologia, Santa Casa de Belo Horizonte, Belo Horizonte, MG, Brasil

**Keywords:** Serum TSH, adults, screening, repetition, 5 years

## Abstract

**Objective:**

To evaluate changes in thyroid function after 5 years, the interval proposed for new assessment, in initially euthyroid adults.

**Subjects and methods:**

Initially, 1,426 apparently healthy adults considered low risk for thyroid dysfunction, were evaluated by measurement of TSH. After 5 years, 1,215 (85.2%) subjects were reevaluated.

**Results:**

After 5 years, four subjects were receiving levothyroxine (L-T4) replacement therapy and 25 others had TSH > 4 mIU/L, only two of them with TSH > 10 mIU/L. All of these subjects had TSH > 3 mIU/L in the initial evaluation. During reassessment, none of the subjects had been or was treated for hyperthyroidism and 22 had TSH < 0.4 mIU/L (none of them < 0.1 mIU/L). Nineteen of these subjects had TSH ≤ 0.6 mIU/L in the initial evaluation. Among the 1,098 subjects with TSH between 0.6 and 3 mIU/L in the initial evaluation, reassessment showed that none of the subjects was using L-T4; only three had TSH > 4 mIU/L (none of them > 10 mIU/L); none had been or was treated for hyperthyroidism, and only three had TSH < 0.4 mIU/L (none of them < 0.1 mIU/L). These results did not differ between men and women or between subjects ≤ 60 and > 60 years.

**Conclusion:**

Repeat TSH measurement within an interval of only 5 years would not be cost-effective in adults without known thyroid disease or risk factors for dysfunction who exhibit TSH between 0.6 and 3 mIU/L.

## INTRODUCTION

Although no consensus exists regarding age, some societies including the American Thyroid Association (1) and Brazilian Society of Endocrinology and Metabolism [SBEM in the Portuguese acronym] (2) recommend screening for thyroid dysfunction by serum TSH measurement in asymptomatic adults (1,2). In the case of individuals that exhibit normal TSH concentrations in the initial evaluation, a new measurement is recommended after 5 years (1–3). This interval was defined more because it is the recommended interval for periodic assessments of general health rather than resulting from a specific analysis of changes in thyroid function (1).

We previously evaluated thyroid function in healthy adults considered low risk for thyroid dysfunction (4,5). These adults were reevaluated after 5 years, the interval proposed for repetition in initially euthyroid adults (1–3), in order to verify changes in thyroid function in these subjects.

## SUBJECTS AND METHODS

This study was a reevaluation of two previous studies that evaluated the TSH reference values for Brazilian population. The initial results of those studies were published previously (4,5). Initially, 2,532 apparently healthy adults (age > 18 years), not including pregnant women, were interviewed (4,5). Of these, 2,327 subjects agreed to participate in the study. Nine hundred and one subjects were excluded because of high-risk factors for thyroid disease or conditions potentially interfering with thyroid function detected upon clinical evaluation (Table 1) (4,5). The final sample consisted of 1,426 apparently healthy adults considered low risk for thyroid dysfunction without known interfering factors (4,5). TSH was obtained in the initial evaluation.

**Table 1:** Inclusion criteria

Absence of thyroid disease, no current or previous treatment with antithyroid drugs or L-T4, no history of ^131^I therapy or thyroidectomy
No use of potentially interfering medications such as dopaminergic agonists or antagonists, neuroleptics, corticosteroids, estrogen, amiodarone, interferon, lithium, anticonvulsants, metformin, octreotide; or recent (in the past 8 weeks) exposure to iodinated contrast agents
No history of head and neck external radiotherapy
Absence of type 1 diabetes or other autoimmune diseases
No family history of thyroid disease
Absence of goiter or any palpable thyroid anomaly
Absence of ophthalmopathy
Normal free T4

After 5 years, these subjects were invited for a new clinical evaluation and TSH measurement. Of the 1,426 subjects included, 1,215 (85.2%) were reevaluated (605 men and 610 women, 915 with age ≤ 60 years and 300 with age > 60 years). Reassessment was not possible in the remaining subjects because of failure to contact them, change of city, and refusal to participate.

The study was prospective and was approved by the local Research Ethics Committee.

Serum samples were obtained from the subjects in the morning (at about 8 a.m.) after an 8- to 10-h fast. TSH was measured with a chemiluminescent assay (Immulite 2000, Diagnostic Products Corporation, Los Angeles, CA), with functional sensitivity of 0.02 mIU/L, and intra- and interassay coefficients of variation < 7% for values ranging from 0.1 to 40 mIU/L.

The Fisher's exact test was used for statistical analysis (comparison between the two groups). A P-value less than 0.05 was considered significant.

## RESULTS

After 5 years, four subjects were receiving levothyroxine (L-T4) replacement therapy initiated because of confirmed subclinical hypothyroidism and 25 others had TSH > 4 mIU/L, only two of them with TSH > 10 mIU/L. It is worth noting that all subjects receiving L-T4 therapy and 22 subjects with TSH > 4 mIU/L (including the two with TSH > 10 mIU/L) had TSH > 3 mIU/L in the initial evaluation.

During reassessment, none of the subjects had been or was treated for hyperthyroidism and 22 had TSH < 0.4 IUI/L (none of them with TSH < 0.1 mIU/L). It is worth noting that 19 subjects with TSH < 0.4 mIU/L had levels ≤ 0.6 mIU/L in the initial evaluation.

Among the 1,098 subjects with TSH between 0.6 and 3 mIU/L in the initial evaluation, reassessment revealed that none of them was receiving L-T4; only three had TSH > 4 mIU/L (none of them with TSH > 10 mIU/L); none of the subjects had been or was treated for hyperthyroidism, and only three had TSH < 0.4 mIU/L (none of them with TSH < 0.1 mIU/L). Among the subjects with initial TSH between 0.6 and 3 mIU/L, the proportions of subjects with TSH of 0.4-4 mIU/L, < 0.4 mIU/L or > 4 mIU/L after 5 years were the same in men (n = 546: 0.18%, 99.45%, and 0.37%, respectively) and women (n = 552: 0.36%, 99.46%, and 0.18%, respectively) and in subjects £ 60 years (n = 822: 0.24%, 99.51%, and 0.24%, respectively) and > 60 years (n = 276: 0.36%, 99.28%, and 0.36%, respectively).

The results of reassessment after 5 years according to initial TSH are shown in Table 2.

**Table 2:** Results of reassessment after 5 years according to initial serum TSH

Initial TSH (mIU/L)	Reassessment after 5 years						
	Treatment for hyperthyroidism	TSH < 0.1 mIU/L	TSH 0.1-0.4 mIU/L	TSH 0.4-4 mIU/L	TSH 4-10 mIU/L	TSH > 10 mIU/L	Under L-T4 therapy
< 0.4 (0.2-0.38) (n = 25)	0	0	13 (52%)	12 (48%)	0	0	0
0.4-0.6 (n = 25)	0	0	6 (24%)	19 (76%)	0	0	0
0.6-3 (n = 1,098)	0	0	3 (0.27%)	1092 (99.45%)	3 (0.27%)	0	0
3-4 (n = 38)	0	0	0	30 (79%)	5 (13%)	1 (2.6%)	2 (5.2%)
> 4* (up to 5.84) (n = 29)	0	0	0	11 (37.9%)	15 (51.7%)	1 (3.4%)	2 (6.9%)

* None of the 24 patients without TPOAb in the initial assessment was treated with L-T4, 11 had normal TSH, and 13 continued to have elevated TSH but < 10 mIU/L. Among the 5 patients with TSH > 4 mIU/L and positive TPOAb in the initial assessment, 2 had elevated TSH < 10 mIU/L, one had TSH > 10 mIU/L, and 2 were taking L-T4.

## DISCUSSION

The results of the present study confirm that in subjects without known thyroid disease, but with a slightly reduced TSH measurement (0.2-0.4 mIU/L), spontaneous TSH normalization in the subsequent measurement is common (6). Even if normalization does not occur, persistence of the initial finding is more common than progression to TSH < 0.1 mIU/L or clinical hyperthyroidism (6,7). The same applies to adults with slightly elevated TSH (4-6 mIU/L) (6,8,9), with the observation of TSH normalization in approximately 40% and persistence of slightly elevated TSH in 50%, while progression to TSH > 10 mIU/L or need for L-T4 after 5 years is observed in only 10%. The consensual recommendation is to repeat the test in these individuals with slightly altered TSH before any treatment decision (10–12). Another implication of this observation is that in population studies evaluating the association between thyroid function and certain outcome(s), but obtaining only a single TSH measurement, a large proportion of the subjects with slightly altered TSH may, in fact, not have true thyroid dysfunction.

The most relevant finding of the study was that, among subjects without known thyroid disease or risk factors for thyroid dysfunction and with initially normal TSH (0.4-4 mIU/L), none had TSH < 0.1 mIU/L and only 0.25% had TSH > 10 mIU/L after 5 years. Most subjects who progressed to reduced (but > 0.1 mIU/L) or elevated TSH (but < 10 mIU/L) had initial TSH < 0.6 and > 3 mIU/L, respectively. With initial TSH between 0.6 and 3 mIU/L, 99.5% of the subjects remained euthyroid after 5 years. Thus, repetition of TSH in this subgroup detected alterations in only one of every 200 retested subjects and even these few individuals with altered TSH did not require treatment. A known previous study demonstrated that 98% of individuals with initially normal TSH continued to present normal TSH when retested within an interval of 5 years (6). However, important limitations of that study were (i) its retrospective design, (ii) only patients with known thyroid disease were excluded but not individuals at high risk for dysfunction, and (iii) the second TSH was obtained on average 19 months after the first measurement (6). Furthermore, that study was conducted exclusively in the Israeli population (6).

We highlight some characteristics of our study. This was a prospective study and the reassessment rate was high (85%). The interval to reassessment was exactly that recommended by some societies (1–3), including SBEM (2). Because this is a current recommendation in Brazil (2), we believe it is important to conduct a study in our country that evaluates this interval for TSH repetition. To our knowledge, no such study exists. On the other hand, it is important to note that the results reported here apply to individuals without known thyroid disease or without the risk factors for dysfunction listed in Table 1. Although measurement of antithyroid peroxidase antibodies (TPOAb) or ultrasonography (US) is not indicated in these individuals, the results could be different if TPOAb are positive or US is altered. The great stability in thyroid function observed after 5 years was limited to subjects with initial TSH between 0.6 and 3 mIU/L. Finally, the results permit us to conclude that the recommended interval of 5 years (1–3) seems to be too short to reevaluate thyroid function, but the appropriate interval cannot be defined and it cannot be stated whether repetition would only be indicated if any clinical suspicion appears.

In conclusion, in this prospective Brazilian study performed in adults without known thyroid disease or risk factors for thyroid dysfunction (Table 1) who exhibit TSH between 0.6 and 3 mIU/L, TSH repetition within the recommended interval of 5 years (1–3) was not cost-effective. Two hundred reassessments were necessary to detect a single case of altered TSH, which even did not result in immediate treatment.
